# The High-efficiency LED Driver for Visible Light Communication Applications

**DOI:** 10.1038/srep30991

**Published:** 2016-08-08

**Authors:** Cihun-Siyong Alex Gong, Yu-Chen Lee, Jyun-Liang Lai, Chueh-Hao Yu, Li Ren Huang, Chia-Yen Yang

**Affiliations:** 1 Department of Electrical Engineering, College of Engineering, Chang Gung University, Taoyuan 33302, Taiwan; 2 Portable Energy System Group, Green Technology Research Center, College of Engineering, Chang Gung University, Taoyuan County 33302, Taiwan; 3 Chang Gung Memorial Hospital at Linkou, Taoyuan 33304, Taiwan; 4 Department of Green Electronics Design and Application, Industrial Technology Research Institute, Hsinchu 31040, Taiwan; 5 Department of Biomedical Engineering, Ming-Chuan University, 5 De Ming Rd., Guishan Township, Taoyuan County 33348, Taiwan

## Abstract

This paper presents a LED driver for VLC. The main purpose is to solve the low data rate problem used to be in switching type LED driver. The GaN power device is proposed to replace the traditional silicon power device of switching LED driver for the purpose of increasing switching frequency of converter, thereby increasing the bandwidth of data transmission. To achieve high efficiency, the diode-connected GaN power transistor is utilized to replace the traditional ultrafast recovery diode used to be in switching type LED driver. This work has been experimentally evaluated on 350-mA output current. The results demonstrate that it supports the data of PWM dimming level encoded in the PPM scheme for VLC application. The experimental results also show that system’s efficiency of 80.8% can be achieved at 1-Mb/s data rate.

The light-emitting diode (LED) has been the environmentally friendly mainstream for lighting applications since its high efficiency and long lifetime were established. The incandescent lamp and fluorescent lamp are going to be replaced by LED gradually. Recently, it has been discovered that LED is more suitable for the high speed communications than the traditional light sources due mainly to its wider modulation bandwidth. This has taken a known technology called the visible light communication (VLC) to its next level^1^.

Research works have been focusing on realizing high data rate transmission for VLC. The Bias-T circuit technique is usually utilized to combine DC driving current for lighting with the data for transmission^2^,^3^. The use of Bias-T and single (multiple) power amplifier(s) is needed on the data path^4^,^5^. However, this causes high cost and large size on the system as well as severe decay in the efficiency. To build VLC technology at existing facility while at the same time making better tradeoffs among cost, speed, and efficiency, this paper presents a LED driver with maximum data rate of 1 Mb/s. Also, 80.8-% system efficiency can be demonstrated at this data rate.

## Proposed VLC LED Architecture

**Figure 1:**
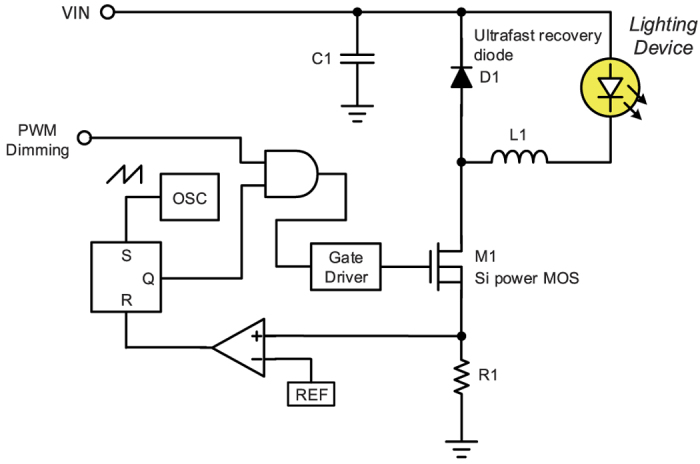
Conventional floating buck LED Driver with pulse width modulation (PWM) dimming.

For the VLC technology, switching type LED driver is a general structure. It not only offers advantage of high efficiency but also great potential in lighting market. To regulate the current of LED with simple and economic method, the conventional structure shown in Fig. 1 possesses floating buck converter with peak current mode control^6^. This structure also has pulse width modulation (PWM) circuit to process illumination dimming. However, the switching type LED driver has limitation of the transmission speed for VLC application. The main reason of such a limitation is its switching frequency limited by switching speed of traditional silicon power device and its significant switching loss. The use of traditional silicon power device results in low switching frequency of the driver, and the frequency is usually slower than several hundreds of kHz. Furthermore, for the purpose of minimizing the current and voltage ripple of LED with such a low switching frequency, large passive device is unavoidable for the energy storage. Unfortunately, the large passive device brings about the lower resonance frequency. On the other hand, the loop bandwidth is usually lower than one tenth of switching frequency for the purpose of stability, and consequently the maximum data rate of switching type LED driver is often limited by several tens of kb/s.

**Figure 2:**
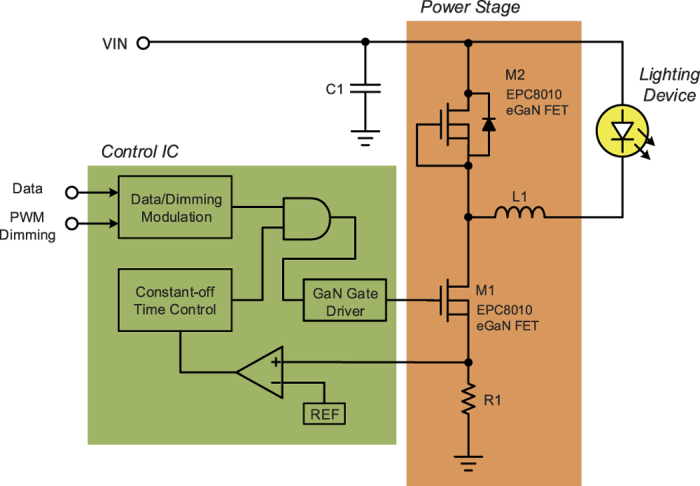
Proposed VLC LED driver.

The proposed VLC LED driver is shown in Fig. 2. To solve the problem of low data rate, GaN power device has been utilized to replace the traditional silicon power device. Thanks to its high switching speed and low switching loss, the proposed driver achieves 10-MHz switching frequency, increasing the bandwidth of data transmission accordingly. The maximum data rate can be up to 1 Mb/s. However, the ultrafast recovery diode, shown in Fig. 1, with large reverse recovery charge (Qrr) results in a long switching interval and high switching loss of low side power MOSFET^7^. The proposed structure includes diode-connected GaN power transistor as a rectifier, shown in Fig. 2. Instead of the traditional ultrafast recovery diode, the diode-connected GaN power transistor M2 featuring zero Qrr has been used to increase the data rate and efficiency of the LED driver, leading to a system’s efficiency of 80.8% at 1-Mb/s data rate. The M2 is OFF when the M1 is ON. Conversely, the M2 is ON while the M1 is OFF.

**Figure 3:**
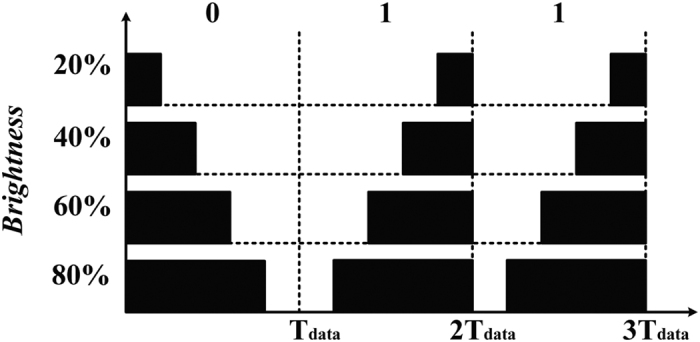
Variable pulse position modulation (PPM) scheme with different PWM dimming levels.

As shown in Fig. 2, the constant off-time controlled loop is used to regulate the LED current stably for wide input voltage. In addition, there is a data/dimming modulation circuit to combine the PPM data with PWM diming signal for VLC and dimming applications^8^. As shown in Fig. 3, the predetermined pulse width is used to decide the dimming level in a modulation period, while at the same time the pulse position is used to determine if bit 0 or bit 1 happens. As the case shown in Fig. 3, the data of bit 0 is represented by the pulse at leading edge of a modulation period. On the other hand, bit 1 is represented by the pulse at trailing edge. The gate driver of control IC with independent push and pull paths is used to prevent overvoltage or overcurrent damage on GaN power transistor. The overvoltage or overcurrent damage is due to high di/dt and dv/dt coming from the parasitic elements of transistor during switching interval^9^.

## Reverse Recovery Effect on System’s Efficiency

**Figure 4:**
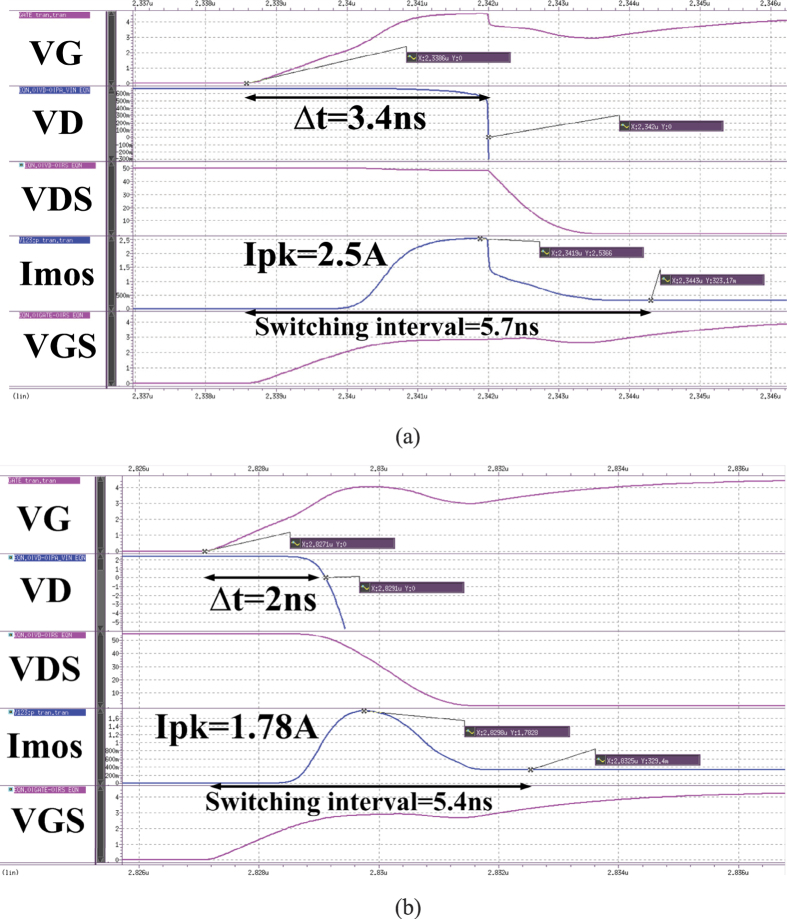
Waveforms at the moment that the lower switch is ON. The LED driver serves as (**a**) a traditional ultrafast recovery diode or (**b**) a diode-connected GaN power transistor as a rectifier.

In this section, we discusses the reverse recovery effect on system’s efficiency. The comparison between the use of the traditional ultrafast recovery diode and that of the diode-connected GaN power transistor in the proposed LED driver will be shown. For the purpose of high efficiency, the lower MOSFET of LED driver should act like a switch. For example, when the power MOSFET of the LED driver is switching on, it requires operation from the cutoff region to saturation region, followed by the triode region. The MOSFET of the LED driver can only stay in the saturation region for a while during switching. That is to say the higher rectifier of the LED driver plays an important role during the process of switching on. As shown in Fig. 4, after the gate-source voltage (VGS) on the lower MOSFET reaches the threshold voltage, the lower MOSFET forms current in the saturation mode, causing increase in the MOSFET current (Imos). Then, common-source inductor of MOSFET resists the change of VGS and slows down the increasing speed of VGS and Imos. The drain-source voltage (VDS) of MOSFET will start to decrease until the reverse recovery charge (Qrr) of diode is depleted completely while the voltage on diode (VD) is turning into zero.

Next, the Imos will decrease to the designed value, followed by the process of switching on becomes complete. As shown in Fig. 4(a), it costs 3.4 ns from the timing of VG turns on to that of VD turns back to 0 V. In Fig. 4(b), it costs 2 ns for it. The results show that the traditional ultrafast recovery diode, which has more Qrr than that of the diode-connected GaN power transistor, takes more time to deplete charges. As a result, the MOSFET will stay long in the saturation mode and the peak current (Ipk) of it will be higher than that of the driver with the diode having lower Qrr. As shown in Fig. 4(a,b), the Ipk at the moment that the lower switch is ON will be 2.5A and 1.78A for the LED driver serving, respectively, as a traditional ultrafast recovery diode and a diode-connected GaN power transistor as a rectifier. To sum up, the LED driver serves as a large Qrr diode as a rectifier will extend the interval of switching and increase the current overshoot on MOSFET. This phenomenon not only results in another switching loss of the LED driver but also brings about possible damage in transistor.

**Figure 5:**
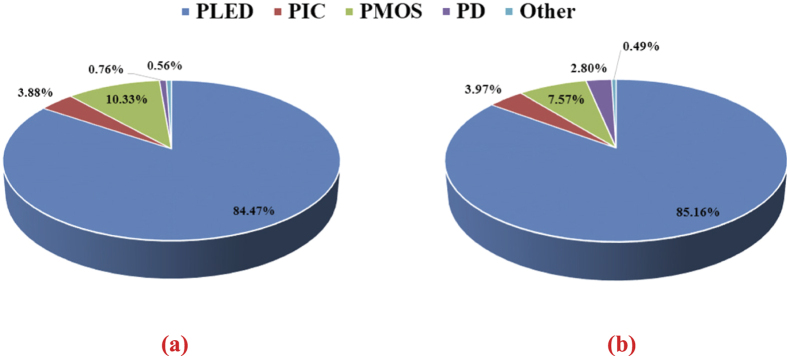
Power distribution of the LED Driver. (**a**) With traditional ultrafast recovery diode. (**b**) With diode-connected GaN power transistor. “PLED” denotes “power dissipation of LED string”. “PIC” denotes “power dissipation of integrated circuit”. “PMOS” denotes “power dissipation of MOS”. “PD” denotes “Power dissipation of diode”.

The simulation results of the power loss distribution in the proposed LED driver are shown in Fig. 5 where a comparison between the use of traditional ultrafast recovery diode and that of the diode-connected GaN power transistor is shown. The simulation was carried out under the conditions 1) the converter is operated at 10 MHz, 2) the input voltage is 53 V, 3) the output voltage is 28.8 V, 4) the LED current is 350 mA with 100-mA ripple, and 5) the VLC input data is 50% PWM dimming level encoded in a 1-MHz PPM. From Fig. 5(a,b), after the use of diode-connected GaN power transistor in the LED driver, the system’s efficiency is increased from 84.47% to 85.16%. The diode and MOSFET are the two items that have the most impact on the power distribution. Although the power distribution of diode is increased by 2.04% from Fig. 5(a,b), the power distribution of MOSFET is decreased by 2.76%. The reason is that the forward voltage of diode-connected GaN power transistor is 2.4 V with 350 mA, and the traditional ultrafast recovery diode’s is 0.67 V. Therefore, although the LED driver serves the diode-connected GaN power transistor as a rectifier will increase the power loss of diode, the decrease in switching loss and current overshoot on MOSFET results in higher system efficiency.

## Experimental Results

**Figure 6:**
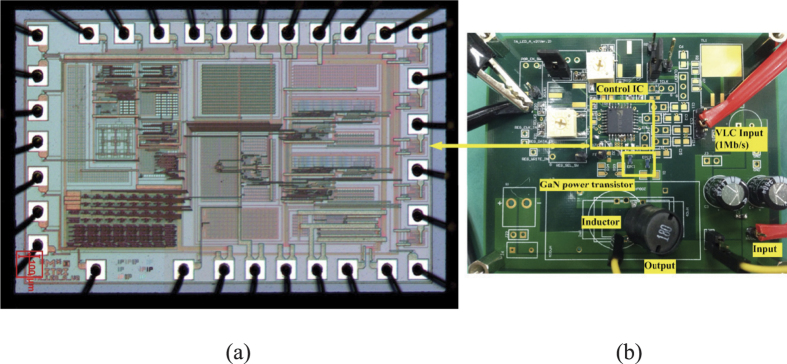
(**a**) Control IC (**b**) LED driver module.

**Figure 7:**
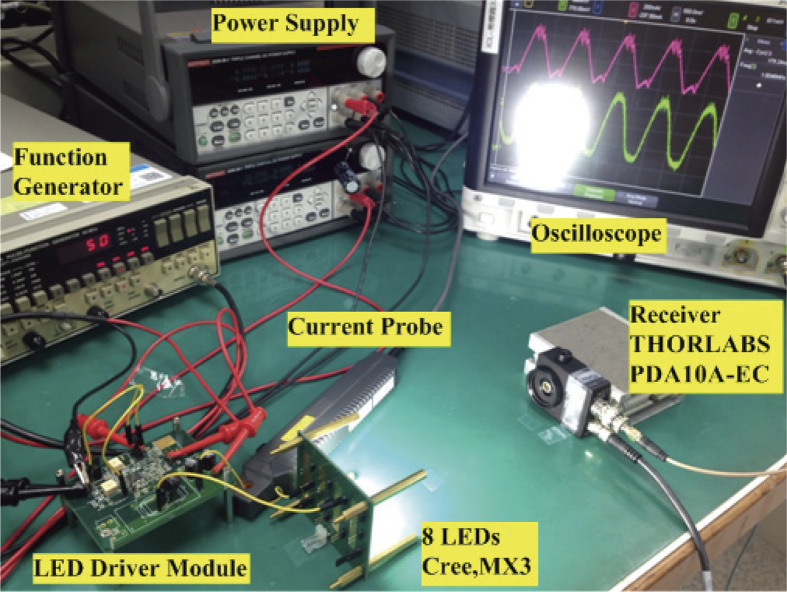
Verification platform of the VLC system.

The proposed VLC LED driver is designed with 350 mA and transmission data rate of 1 Mb/s. The converter is operated at 10 MHz with the input voltage of 50 V and output voltage of 25 V. The data of VLC used in the measurement is a 50-% PWM dimming level encoded in 1-MHz PPM. The control IC is shown in Fig. 6(a) where it was fabricated in a standard 0.5-μm CMOS technology. It measures 1930 μm × 1250 μm. A standard SOP-32 pin package has been used for measurement. The LED Driver module is shown in Fig. 6(b) where its discrete components involve diode-connected GaN power transistor (EPC, EPC8010), GaN power transistor (EPC, EPC8010), and 18-μH inductor. The verification platform is shown in Fig. 7. The power supply is used to light up the LED, and the signal of 50-% PWM dimming level encoded in 1 MHz PPM is input through the VLC input pin of the LED driver module. The current of LED and receiver was measured by current probe and oscilloscope.

**Figure 8:**
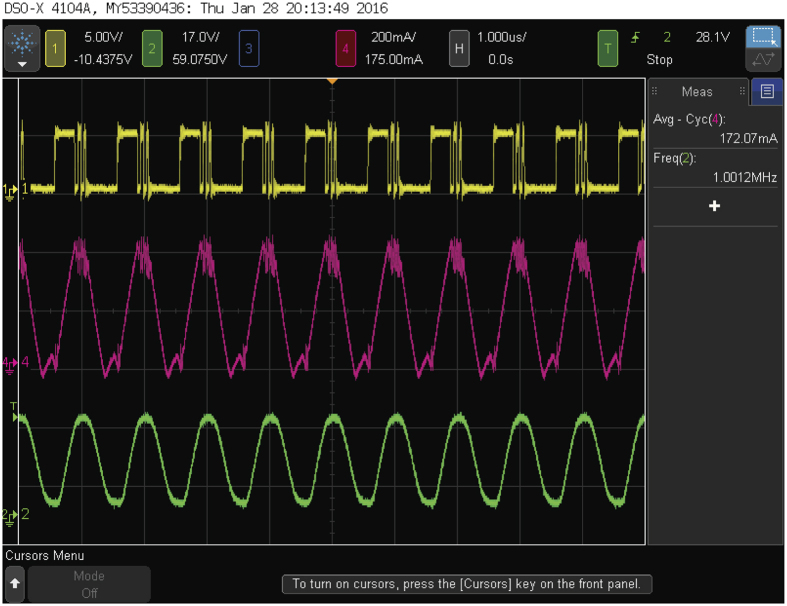
Experimental waveforms. From top to bottom are (**a**) Gate voltage of the lower GaN power transistor, (**b**) LED current, and (**c**) Receiver current.

As shown in Fig. 8, the waveforms from top to bottom are the gate voltage of the lower GaN power transistor in the proposed LED driver, the LED current, and the receiver current, respectively. The measurement results show that the average current of LEDs is 172 mA, and consequently the 50% PWM dimming is achieved. In addition, the waveforms of the LED and receiver current show that the data rate of 1 Mb/s has been demonstrated, while at the same time having measured power consumption of 5.68 W and 4.59 w for the system and the LED, respectively. The efficiency of the whole system is 80.8%. As a result, a LED driver with high data rate and high efficiency has been realized. Comparison between the proposed design and state-of-the-art works is tabulated in Table 1. The work presented in ref. 4 utilizes multiple-resonant equalization and blue filter to achieve a transmission rate of 80 Mb/s. The advantage of its design is high transmission rate. However, it requires integrating extra T Bias-tee and multiple power amplifiers with the LED driver, not only increasing overall cost but also decreasing energy conversion efficiency. The work presented in ref. 10 utilizes shunt switch technique to transmit data. It achieves both high transmission rate and high energy conversion efficiency. The data rate can be as high as Mb/s class without being limited by the switching frequency of LED driver. However, it needs an extra power transistor as the shunt switch. The addition of power-transistor-associated gate driver is also unavoidable. As a result, both cost and power are compromised. In our work, we have utilized the GaN power devices as replacements of not only a silicon power device but also an ultrafast recovery diode for switching. Our design can be an effective way to achieve Mb/s-class transmission. Compared with the works in refs 4 and 10, our proposed work integrates both the DC lighting driving signal with the data signal to drive a single power transistor. By taking the advantage, the visible light communication technique can be built directly on the existing indoor illumination infrastructure without additional devices and energy loss, thereby offering higher energy conversion efficiency.

## Conclusion

The proposed LED driver in this paper solves the low data rate problem of conventional switching type LED driver. It is designed for 350 mA output current and supports the data of PWM dimming level encoded in PPM scheme for VLC application. In this paper the GaN power device is used to increase the switching frequency of the LED driver, and consequently the bandwidth of the LED driver can be extended and the data rate can be increased. Moreover, it has been demonstrated that the proposed LED driver with the diode-connected power transistor has higher efficiency than the LED driver with traditional ultrafast recovery diode. The measurement results show that the proposed LED driver achieves 80.8% efficiency under 1-Mb/s data rate. Commercial white (fluorescent powder) LEDs have about 1 MHz~2 MHz modulation bandwidth. The bandwidth is mainly limited by the inherent light emission mechanism. As a matter of fact, despite higher bandwidth in the design of transmitter, the modulation bandwidth is still limited by the LED itself during the electrical-to-optical conversion for visible light communication. To solve the problem, a blue light filter can be added to the front of the receiver front end to remove the yellow light with longer response time. The filter can be an effective way to achieve higher bandwidth, thereby achieving higher switching frequency of the driver in the proposed design. The higher the switching frequency, the higher the data rate.

## Additional Information

**How to cite this article**: Gong, C.-S. A. *et al.* The High-efficiency LED Driver for Visible Light Communication Applications. *Sci. Rep.* **6**, 30991; doi: 10.1038/srep30991 (2016).

## Figures and Tables

**Table 1:** Comparison between the proposed design and state-of-the-art works.

	**This Work**	4	10	**Phillips US8150269**	**Panasonic US20130015784**
Max. Date Rate	1 Mb/s	80 Mb/s	2 Mb/s	<10 kb/s	4 kb/s
Efficiency	81.5% (85.8%)	—	74.6%	—	—
Architecture	Switched-mode w/GaN device	Multiple-resonant equalization w/blue filler	Switched-mode	Switched-mode	Switched-mode
